# Two new species of the genus *Leenurina* Najt & Weiner, 1992 (Collembola, Neanuridae, Caputanurininae) from Primorskij Kraj (Russia)

**DOI:** 10.3897/zookeys.115.1464

**Published:** 2011-07-05

**Authors:** Louis Deharveng, Anne Bedos, Wanda Maria Weiner

**Affiliations:** 1 UMR7205 du CNRS, CP50, Muséum National d’Histoire Naturelle, 45 rue Buffon, 75005 Paris, France; 2 Institute of Systematics and Evolution of Animals, Polish Academy of Sciences, Sławkowska 17, Pl – 31 - 016 Kraków, Poland

**Keywords:** Taxonomy, chaetotaxy, integument granulation, Eastern Asia

## Abstract

*Leenurina khualaza* **sp. n.** and *Leenurina pomorskii* **sp. n.**, two new species from East Russia (Primorskij Kraj) are described. They are closely related to *Leenurina jasii* Najt & Weiner, 1992 from North Korea, from which they differ mainly in the number of tibiotarsal chaetae (19, 19, 18 in the new species versus 18, 18, 17 in *Leenurina jasii*), several chaetotaxic features and organization of dorsal granulation. The two new species may be separated by tertiary granulation (large areas fringed with large secondary granules in *Leenurina pomorskii*, small rounded or hexagonal areas with smaller secondary granules in *Leenurina khualaza*), coloration (light blue in *Leenurina khualaza* versus white in *Leenurina pomorskii*) and number of eyes (2+2 eyes in *Leenurina khualaza* versus 3+3 eyes in *Leenurina pomorskii*). An updated diagnosis of the genus *Leenurina* Najt & Weiner, 1992 and a key to species of Caputanurininae are given.

## Introduction

Caputanurininae is a small subfamily of Neanuridae defined by a remarkable synapomorphy, unique among Collembola: the fusion of prothoracic tergite to head. It includes two genera: *Caputanurina* Lee, 1983 and *Leenurina* Najt & Weiner, 1992. *Koreanurina* Najt & Weiner, 1992, assigned to Pseudachorutinae, is very similar to these genera, but its prothorax is separated from head (Najt and Weiner 1992). These three genera represent different degrees in head-prothorax fusion, from separate to completely fused, challenging the validity of the subfamily Caputanurininae as currently defined.

Najt and Weiner (1992) established the genus *Leenurina* for two species: *Leenurina jasii* Najt & Weiner, 1992 from North Korea as type species, and *Caputanurina nana* Lee, 1983 from South Korea. This genus is closely related to *Caputanurina* Lee, 1983, the nominal genus of the Far Eastern subfamily Caputanurininae. Differences between these two genera are summarized in the Table 2 of Najt and Weiner (1992: 204) and in Table 1 of the present paper.

**Table 1.** Differential characters between *Leenurina*, *Caputanurina* and *Caputanurina intermedia*.

	*Leenurina*	*Caputanurina*	*Caputanurina intermedia*
Eye position	dorsal	dorso-lateral or lateral	dorso-lateral
PAO position	dorsal	latero-ventral or ventral	lateral
Abd. IV-V suture	as shallow inverted V	as deep inverted V	as deep inverted V
Habitus	slightly flattened	strongly flattened	flattened
Number of chaetae between the anterior line of chaetae and the posterior margin of the central plate on head	1-3+1-3<br/> in one or two rows	>3+3<br/> in several rows	3+3<br/> in two rows
Number of chaetae p between axis and S on abd. II-III	2	3	3

Caputanurininae and the related genus *Koreanurina* are only known for temperate regions of Far-East Asia, i.e. South Korea (Lee 1983), North Korea (Najt and Weiner 1992), and northeastern China (Wu and Yin 2007).

Among a large material of Collembola collected in Primorskij Kraj, two new representatives of the genus *Leenurina* were found. They are described in this paper, and led us to correct and complete the diagnosis of this genus. They are the first Caputanurininae described from Russia, though an unidentified species of *Caputanurina* was already mentioned from Far East Russia – South Primorie (Kuznetsova 1988).

### Abbreviations used

MNHNMuséum national d’Histoire naturelle de Paris (France)

ISEAInstitute of Systematics and Evolution of Animals, Polish Academy of Sciences, Kraków (Poland)

DBETDepartment of Biodiversity and Evolutionary Taxonomy, Wrocław University (Poland)

MSPUMoscow State Pedagogical University, Moscow (Russia)

## Material and methods

The specimens were extracted from forest litter samples using Berlese funnels, and stored in 90% ethanol. They were cleared in lactic acid, mounted on slide in Marc-André II and examined using a microscope Leica DMLB. Photographs were taken with a ProgRes C3 camera mounted on the microscope, using either phase contrast (Fig. 6) or DIC interferential contrast (Figs 2A, 4, 5). Chaeta numbering in the text and figures follows Yosii (1960) and Cassagnau (1974).

Abbreviations used in tables and key: abd., abdominal tergum; PAO, postantennal organ; th., thoracic tergum; ant., antennal segment;.

## Systematics

**Neanuridae**

**Caputanurininae**

### Leenurina

Najt & Weiner, 1992

http://species-id.net/wiki/Leenurina

#### Type species:

*Leenurina jasii* Najt & Weiner, 1992

#### Diagnosis.

Body wide, flattened dorso-ventrally. Thoracic tergum I fused to head. Suture between abdominal tergum IV and V normal or as a shallow inverted V. Integument strongly granulated dorsally, with tertiary granulations variously arranged and underlying of hexagonal regular reticulations on head, thorax II and III, and abdomen I to V. Eyes and postantennal organ located dorsally. Postantennal organ made of 9-14 entire vesicles in one row. Mandibles with five teeth, maxillae thin. Labial organite x present. Papillated chaeta L absent on labium. Antenna with distinct apical vesicle. Antenna IV with 6 thickened sensilla and one microsensillum dorsally. Dorsal chaetotaxy of short and pointed ordinary chaetae and thin s-chaetae. Chaetal arrangement strongly disrupted on head, with a large central area devoid of chaetae. Dorsal chaetotaxy reduced. Claw toothless. Furca reduced to two small swellings, each with one chaeta.

#### Discussion.

On thoracic terga, p1 correspond to chaeta m1 of Najt and Weiner 1992, and p2 to p1. As a result, the s-chaeta is assumed to be in p4 (p5 as in Najt and Weiner 1992). *Leenurina* differs from *Caputanurina*, the other genus of the subfamily Caputanurininae, by the characters listed in Table 1. *Caputanurina intermedia* Najt & Weiner, 1992 exhibits intermediate characters between the two genera, which are closely related.

#### List of species

*Leenurina nana* (Lee, 1983) – South Korea (Gang-weon-do province);

*Leenurina jasii* Najt & Weiner, 1992 – North Korea (Kangwon and North Hamgyong provinces), type species of the genus;

*Leenurina khualaza* sp. n. – Russia (Primorskij Kraj);

*Leenurina pomorskii* sp. n. – Russia (Primorskij Kraj).

### Leenurina khualaza sp. n.

urn:lsid:zoobank.org:act:905C5771-F476-4371-80C5-20A16D3AFD8F

http://species-id.net/wiki/Leenurina_khualaza

**Figure 1.**
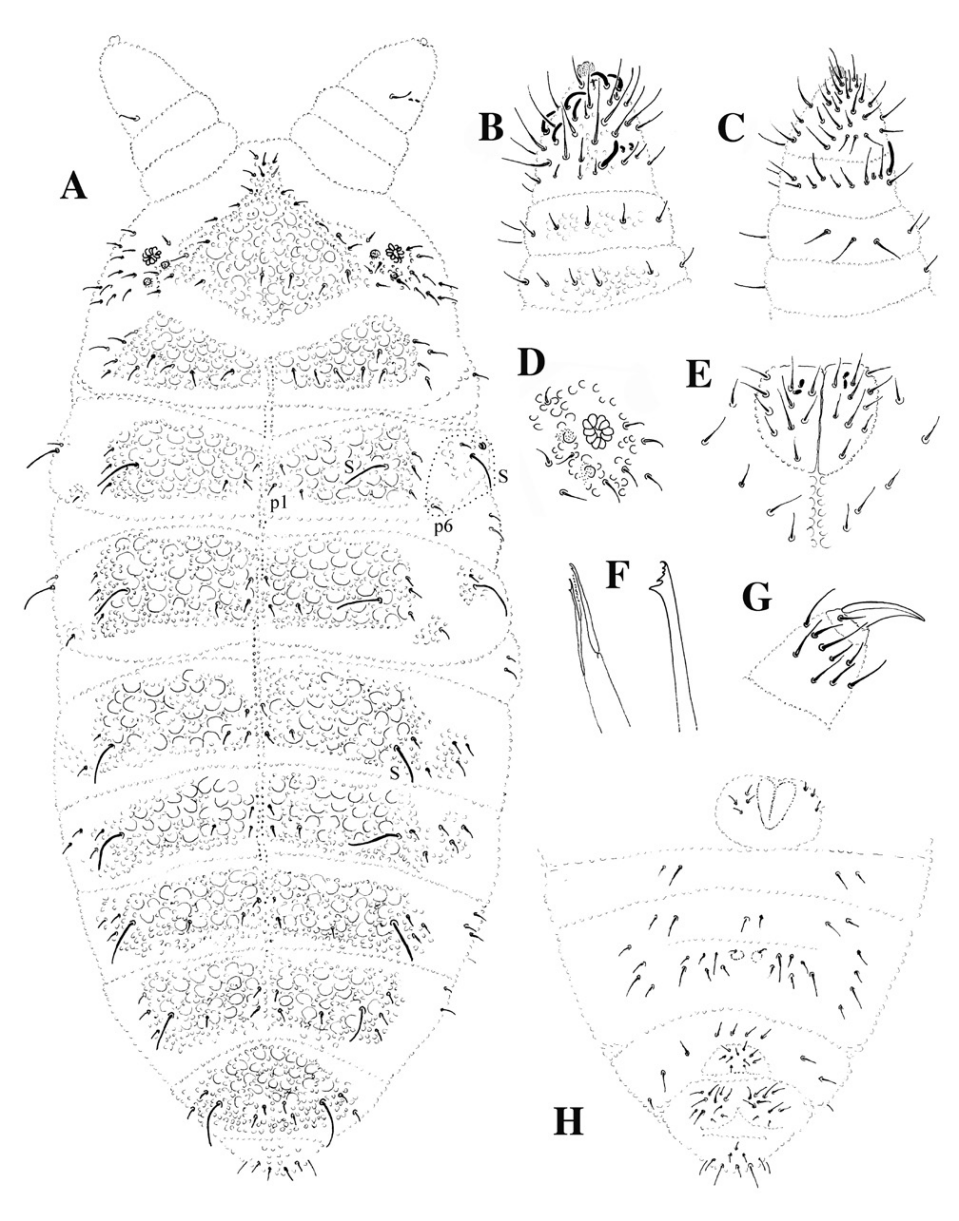
*Leenurina khualaza* sp. n.; **A** dorsal chaetotaxy (dorso-lateral chaetae circled with dotted line) **B** antenna, dorsal side **C** antenna, ventral side **D** postantennal organ and ocelli **E** labium **F** maxilla (left) and mandible (right) **G** tibiotarsus III **H** chaetotaxy of abdominal sterna I–VI.

Figs 1A–H 6C, D, H Table 2 

#### Type locality.

Russia: Primorskij Kraj, Shkotovsky area, Livadiysky Range, Anisimovka (43° 10’ 11” North, 132° 47’ 37” East), Khualaza Mt. Litter in mixed deciduous and coniferous forest, Berlese funnel extraction, L. Deharveng and A. Bedos leg, 19.IX.04 (samples RU-032, RU-031, RU-029).

#### Type material.

Holotype, female adult (RU-032/1) and 7 paratypes, on slides. Holotype and 1 paratype male adult (RU-032/5) in MNHN; 2 paratypes: female (RU-032/2), male juvenile (RU-031) in ISEA; 2 paratypes: female (RU-032/3), male juvenile (RU-032/4) in DBET; 2 paratypes: females (RU-032/6, RU-029) in MSPU.

#### Etymology.

After the name of the type locality, the Khualaza mountain.

#### Description.

Holotype: 0.70 mm (female adult); paratypes: 0.60–0.70 mm (females), 0.45 (male RU032/5) to 0.61–0.62 mm (males juvenile). Habitus typical for the genus *Leenurina*. Abdominal tergum VI small, not hidden under V. Color in alcohol very light blue with blue-black 2+2 ocelli. Integument very strongly granulated dorsally, with tertiary granulation arranged in rather small and smooth subhexagonal areas encircled by 5 to 9 secondary granules, underlined by strong reticulations, and grouped as large plates on head (Figs 6C, H), on thorax II-III and on abdomen I to V (Fig. 6D). Two parallel lines of secondary granules along the axis from posterior part of head to abdominal tergum IV. Thoracic tergum I fused with head, sternum normal.

Antennae shorter than head. Antennal segment I with 7 chaetae, antennal segment II with 12 chaetae. Sensory organ on antennal segment III consisting of two small sensilla bent in the same direction, two almost equal, subcylindrical guard sensilla and a small ventral microsensillum. Antennal segment IV with 6 thick subcylindrical sensilla, a microsensillum, a subapical organite and a simple apical vesicle (Figs 1B, C).

Two ocelli per side, a little larger than surrounding integument granulation, indicated by blue-black pigment patches, but not distinct from surrounding secondary granules under microscopic examination. Postantennal organ slightly oval, about three times longer and two times broader than ocellus A, with 9–10 vesicles (Figs 1A, D). Buccal cone typical for the genus. Labrum truncated, labral chaetotaxy: 4/2,3,5,2, with prelabral chaetae as 2 axial and 2 lateral; the later assigned here to labrum might be as well lateral labial chaetae. Labium short, with 4 basal (E, F, G, f), 3 distal (A, C, D) and 3 lateral chaetae; papillated chaeta L absent; 2+2 hyaline vesicles arranged one above the other between chaetae A and C (x papillae of Deharveng 1983) (Fig. 1E). Mandible with three small apical teeth and two strong basal ones. Maxilla with two lamellae (each with two apical teeth) and capitulum denticulate with minute teeth (Fig. 1F).

Dorsal chaetotaxy as on Fig. 1A, with thin short pointed ordinary chaetae and long thin s-chaetae, 4–5 times longer than ordinary chaetae. Some asymmetry observed. Ocular area with 3 chaetae. One lateral chaeta (Fig. 1A) located on what could be the subcoxa 1. Dorso-lateral chaetae of thoracic terga II and III in two groups (p6 shift posteriorly far from the s-chaeta, Fig. 1A). Formula of s-chaetae per half tergum: 022/11111; s-microchaeta present on thoracic tergum II, close and anterior to the lateral s-chaeta; s-chaeta on abdominal tergum IV almost as long as on abdominal terga II and III. From thoracic tergum II to abdominal tergum IV, 3 chaetae between the axis and the proximal s-chaeta: a1, p1 and a chaeta moving from a “p2” (usually on thoracic tergum II to abdominal tergum II) to a “p3” position (usually on abdominal tergum III-IV), with variation from one specimen and sometimes one side to the other.

Thoracic sterna without chaetae. Chaetotaxy of abdominal sterna I–VI as in Fig. 1H. Lateral anal valves with two, upper valve with three hr-chaetae.

Tibiotarsi I, II and III with 19, 19 and 18 chaetae (chaeta M present). Femora I, II and III with 12, 11 and 10 chaetae, trochantera I, II and III each with 5 chaetae, coxae I, II and III with 3, 6 and 7 chaetae, subcoxae 2 of legs I, II and III with 0, 1 and 1 chaetae, subcoxae 1 of legs I, II and III with 1, 2 and 2 chaetae. Praetarsi with 1+1 strong chaetae. Claw short and thick, toothless (Fig. 1G).

Ventral tube with 4+4 chaetae, without chaetae at its basis. Furca reduced to two small swellings, each with one chaeta (Fig. 1H).

#### Discussion.

See the discussion of *Leenurina pomorskii* and Table 2.

**Table 2.** Differential characters between *Leenurina* species.

Characters	*Leenurina nana* (after original description)	*Leenurina jasii*	*Leenurina khualaza* sp. n.	*Leenurina pomorskii* sp. n.
Colour	orange alive	light blue in alcohol	very light blue in alcohol	white in alcohol
Dorsal tertiary granulation	?	grouping of 2-3 secondary granules	subhexagonal areas encircled by 5 to 9 secondary granules	very large plates fringed with secondary granules
Number of ocelli	2+2	3+3	2+2	3+3
Number of vesicle in PAO	11 in circle	11-14, oval	9-10, slightly oval	10-12, oval
Chaeta p6 and dorso-lateral s-chaeta on th.II–III	?	grouped	widely separate	grouped
Number of chaetae between the anterior line of chaetae and the posterior margin of the central plate on head	2+2 in one row	1-2+1-2 in one row	2+2 in one row	3+3 in two rows
Number of chaetae in the posterior row between s-chaetae of abd. IV	6	4	4	2
Number of chaetae on tibiotarsi I, II, III	18,18,17	18,18,17	19,19,18	19,19,18
Number of chaetae on subcoxae 2 of legs I, II, III	?	0,1,1	0,1,1	0,2,2

### Leenurina pomorskii sp. n.

urn:lsid:zoobank.org:act:5AAA02CB-3C05-4409-8592-240AF93AE8EE

http://species-id.net/wiki/Leenurina_pomorskii

**Figure 2.**
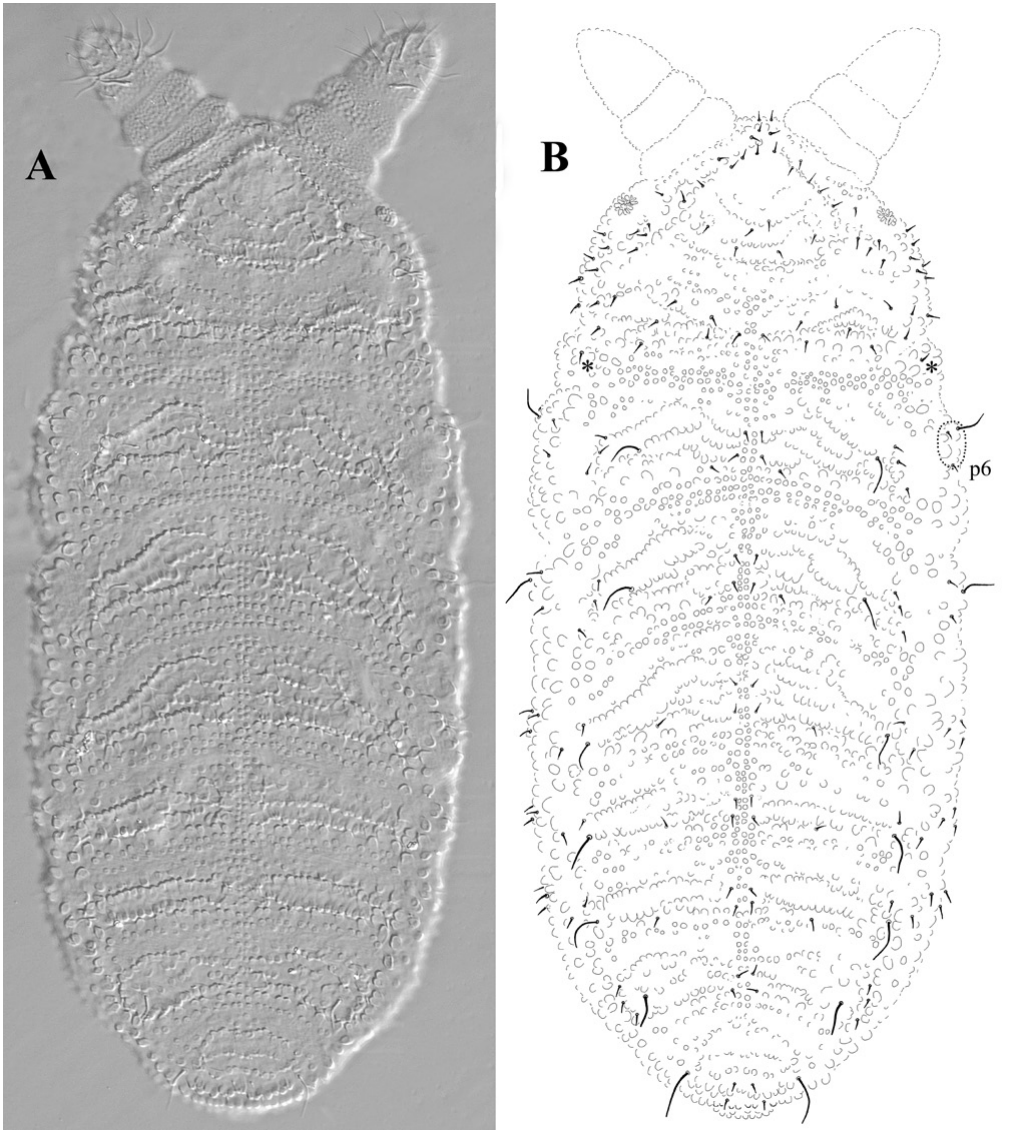
*Leenurina pomorskii* sp. n. in dorsal view; **A** photo Leica DIC **B** tubercles and chaetotaxy. *, lateral chaeta of the first thoracic tergum; dorso-lateral chaetae circled with dotted line.

Figs 2A–B 3A–F 4 5 6E, F, I Table 2 

#### Type localities.

Russia: Primorskij Kraj, Khasan Region, Pos’et Bay, Point Mramornyj (42° 34’ 16” N, 130° 47’ 27” E), litter in mixed deciduous forest, Berlese funnel extraction, 28.IX.04, 5 specimens, L. Deharveng and A. Bedos leg. (sample RU-120).Russia: Primorskij Kraj, Khasan Region, ~ 5 km E of Mayachnoye, Gora Chertova Gorka (42° 37’ 02” N, 130° 42’ 31” E), in mixed deciduous forest, Berlese funnel extraction, 28.IX.04, 3 specimens, R. J. Pomorski leg. (sample 3a).

#### Type material.

Holotype: female adult on slide (RU-120/2) in MNHN. Paratypes: "one female juvenile on slide (RU-120/1) in MNHN; one female juvenile (RU-120/3) and one juvenile (RU-120/4, skin obtained after DNA extraction for barcoding) on slide in ISEA; one female juvenile (3a/1) and one juvenile (3a/3) on slides in DBET; one female (RU-120/5) and one juvenile (3a/2) on slides in MSPU.

#### Etymology.

The new species is dedicated to Professor R. Jacek Pomorski, the eminent taxonomist of Collembola and our friend, who left us in 2010.

#### Description.

Holotype: 0.92 mm (female adult); paratypes: 0.84 mm (female), 0.6-0.9 mm (female juvenile), 0.58 mm (juvenile). Habitus typical for the genus *Leenurina*. Abdominal tergum VI small, sometimes hidden under V (Fig. 2A). Color in alcohol white with 3+3 blue-black ocelli. Integument very strongly granulated dorsally, with tertiary granulation arranged in large smooth plates fringed with lines of strong secondary granules (Figs 2A, B, 6E, F, I). Well marked underlying small hexagonal reticulations (Fig. 5), each reticulation mesh connected with two or three secondary granules. Secondary granules rounded, the lateral ones very large. Two parallel lines of secondary granules along the axis from posterior part of head to abdominal tergum IV. Thoracic tergum I fused with head, sternum normal.

**Figure 3.**
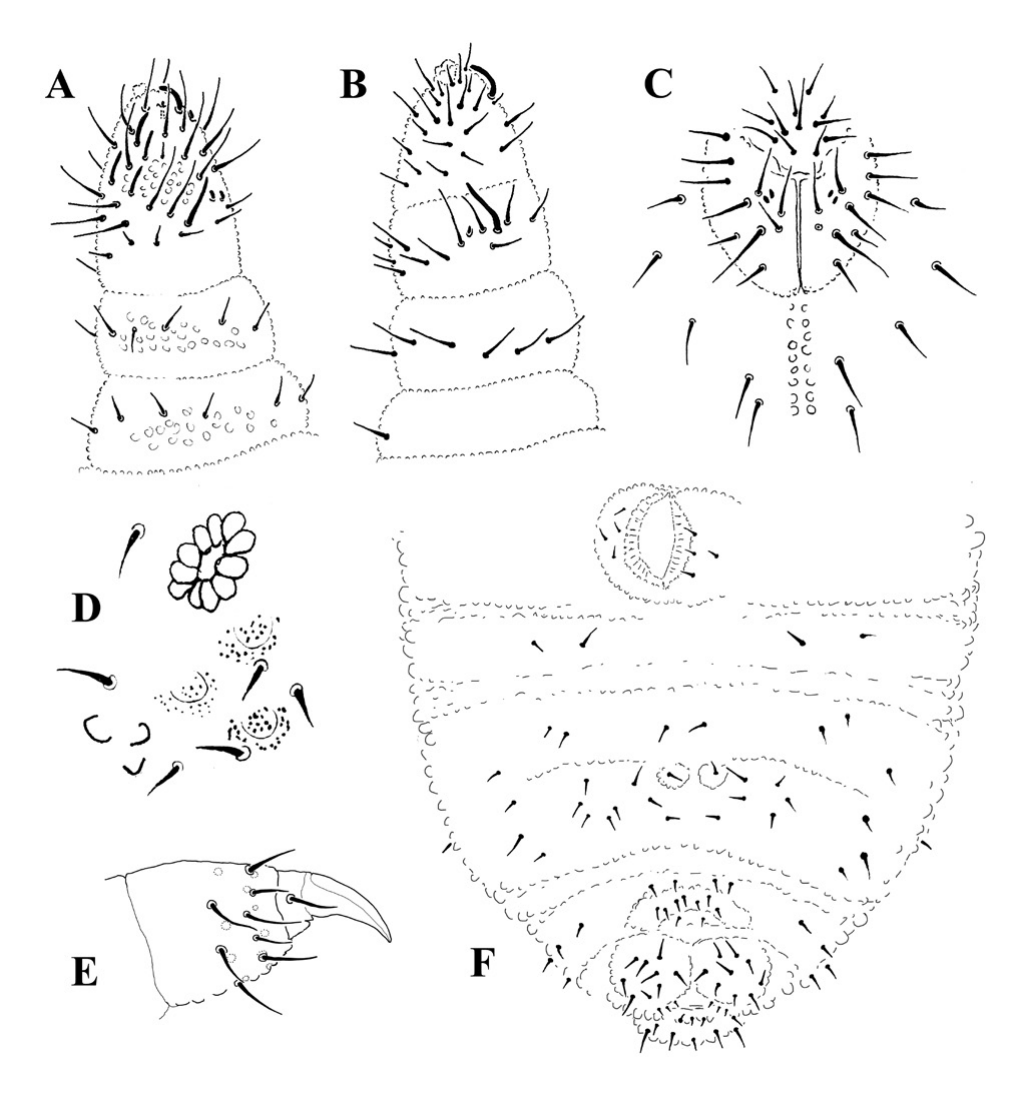
*Leenurina pomorskii* sp. n.; **A** antenna, dorsal side **B** antenna, ventral side **C** labrum and labium **D** postantennal organ and ocelli **E** tibiotarsus III **F** chaetotaxy of abdominal sterna I–VI.

Antennae shorter than head. Antennal segment I with 7 chaetae, antennal segment II with 12 chaetae. Sensory organ on antennal segment III consisting of two small sensilla bent in the same direction, two almost equal, subcylindrical guard sensilla and a small ventral microsensillum. Antennal segment IV with 6 thick subcylindrical sensilla, a microsensillum, a subapical organite and a very slightly bilobed apical vesicle (Figs 3A, B).

**Figure 4.**
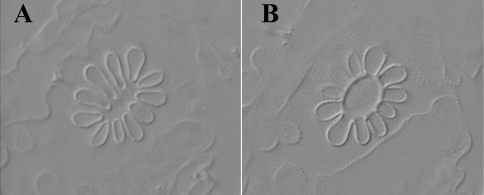
*Leenurina pomorskii* sp. n.; **A** postantennal organ of a same specimen, at two different focus (**A**, surface; **B**, deeper).

Three ocelli per side, slightly bigger than surrounding integument granulation, indicated by blue-black pigment patches, but not distinct from surrounding secondary granules under microscopic examination. Postantennal organ oval, 2-3 times longer and about 1.5-2 times broader than ocellus A, with 10-12 vesicles (Figs 3D, 4). Buccal cone typical for the genus. Labrum truncated, 1-2 prelabral chaetae, labral chaetotaxy uncertain, probably: ?2,3,5,2. Labium short, with 4 basal (E, F, G, f), 3 distal (A, C, D) and 3 lateral (c, d, e) chaetae; papillated chaeta L absent; 2+2 small hyaline vesicles arranged one above the other between chaetae A and C (x papillae of Deharveng 1983) (Fig. 3C). Mandible with three small apical teeth and two strong basal ones. Maxilla with two lamellae (each with two apical teeth) and capitulum denticulate with minute teeth like *Leenurina khualaza*.

Dorsal chaetotaxy as on Fig. 2, with short thin pointed ordinary chaetae and long thin s-chaetae, 3–5 times longer than ordinary chaetae. Some asymmetry observed. Ocular area with 3 (or 4 chaetae, probably as a result of a shift of a dorsal cephalic chaeta towards ocular area). One lateral chaeta on what could be the poorly individualized lateral part of thoracic tergum I (Fig. 2B), and another ventro-lateral chaeta on subcoxa 1. Dorso-lateral chaetae of thoracic terga II and III in one group (p6 close to s-chaeta, Fig. 2B). Formula of s-chaetae per half tergum: 022/11111; s-microchaeta present on thoracic tergum II, close and anterior to the lateral s-chaeta; s-chaetae slighly thicker and shorter on abdominal tergum IV than on other terga. From thoracic tergum II to abdominal tergum III, 3 chaetae present between the axis and the proximal s-chaeta: a1, p1 and a chaeta in a “p2” position on thoracic tergum II-III and usually a “p3” position abdominal terga I-III. Abdominal tergum IV with only 2+2 chaetae between the axis and the proximal s-chaeta (a1, p1). Some specimens slightly depart from this pattern on details: one specimen with 4 chaetae present between the axis and the proximal s-chaeta on abdominal tergum I (a1, p1 and 2 other chaetae in row p) ; one specimen with 2 chaetae present between the axis and the proximal s-chaeta on abdominal tergum I (a1 and p1); one specimen with an additional chaeta antero-internal and close to the s-chaeta on abdominal tergum IV.

Thoracic sterna without chaetae. Chaetotaxy of abdominal sterna I–VI as in Fig. 3F. Anal valves with three hr-chaetae each.

Tibiotarsi I, II and III with 19, 19 and 18 chaetae (chaeta M present). Femora I, II and III with 13, 12 and 11 chaetae, trochantera I, II and III with 6, 6 and 5-6 chaetae, coxae I, II and III with 3, 6 and 7 chaetae, subcoxae 2 of legs I, II and III with 0, 2 and 2 chaetae, subcoxae 1 of legs I, II and III with 1, 2 and 2 chaetae. Praetarsi with 1+1 strong chaetae. Claw short and thick, toothless (Fig. 3E).

Ventral tube with 4+4 chaetae, without chaetae at its basis. Furca reduced to two small swellings, each with one chaeta (Fig. 3F).

#### Discussion.

Dorsal chaetotaxy of both described species exhibits some variability and frequent asymmetries. The four species of *Leenurina* are closely related, but easily distinguished on a combination of characters including eye number, pigmentation, leg and dorsal chaetotaxy (Table 2).

**Figure 5.**
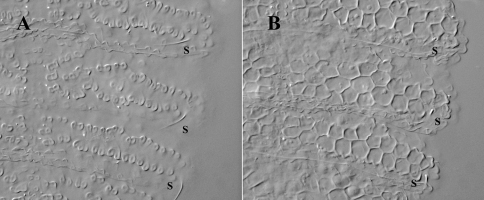
*Leenurina pomorskii* sp. n.; **A** abdominal terga II and III at two different focus in a same specimen (**A**, surface showing tertiary plates; **B**, deeper showing underlying reticulations).

**Figure 6.**
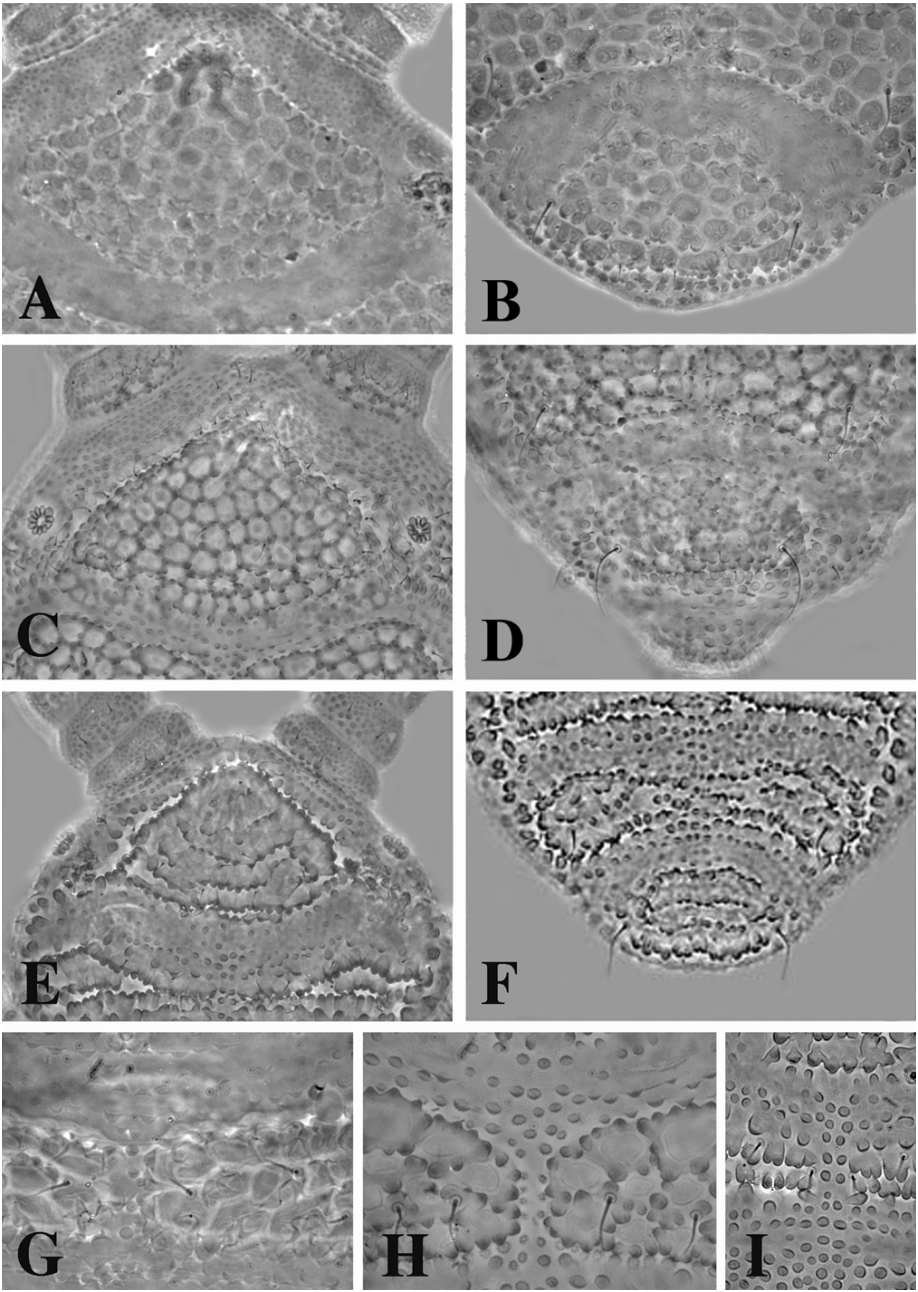
Central area of the head; **A** *Leenurina jasii* Najt & Weiner, 1992 **C** *Leenurina khualaza* sp. n. **E** *Leenurina pomorskii* sp. n. Abdominal terga IV–VI **B** *Leenurina jasii* Najt & Weiner, 1992 **D** *Leenurina khualaza* sp. n. **F** *Leenurina pomorskii* sp. n. Posterior part of head **G** *Leenurina jasii* Najt & Weiner, 1992 **H** *Leenurina khualaza* sp. n. **I** *Leenurina pomorskii* sp. n.

## Key to species of the subfamily Caputanurininae (after Wu and Yin 2007 pro parte)

**Table:** 

1	Eyes and PAO in dorsal position, body slightly flattened	2 *Leenurina* Najt & Weiner, 1992
–	Eyes and PAO in lateral or dorso-lateral position, body flattened or strongly flattened	5 *Caputanurina* Lee, 1983
2	Tibiotarsi I–III with 18, 18, 17 chaetae	3
–	Tibiotarsi I–III with 19, 19, 18 chaetae	4
3	2+2 eyes, abd. IV with 6 chaetae in the posterior row between s-chaetae, orange alive	*Leenurina nana* Lee, 1983; South Korea
–	3+3 eyes, abd. IV with 4 chaetae in the posterior row between s-chaetae, light blue in alcohol	*Leenurina jasii* Najt & Weiner, 1992; North Korea
4	2+2 eyes, abd. IV with 4 chaetae in the posterior row between s-chaetae, dorsal tertiary tubercles small, hexagonal or rounded, very light blue in alcohol	*Leenurina khuazala* sp. n.; Russia, Primorskij Kraj
–	3+3 eyes, abd. IV with 2 chaetae in the posterior row between s-chaetae, dorsal tertiary tubercles as fringed areas, white in alcohol	*Leenurina pomorskii* sp. n.; Russia, Primorskij Kraj
5	2+2 eyes, PAO in latero-ventral or ventral position	6
–	3+3 eyes, PAO in dorso-lateral position, tibiotarsi I–III with 18, 18, 17 chaetae, mandible with 5 teeth	*Caputanurina intermedia* Najt & Weiner, 1992; North Korea
6	Abd. III and IV separate, PAO with 8–12 vesicles	7
–	Abd. III and IV fused, PAO with 13–14 vesicles, on head 3+3 dorso-medial chaetae, vestigial furca with 1+1 chaetae	*Caputanurina sinensis* Wu & Yin, 2007; China, prov. Liaoning
7	Claw without teeth	8
–	Claw with interno-lateral tooth, mandible with 5 teeth, tibiotarsi I–III with 18, 18, 17 chaetae	*Caputanurina turbator* Najt & Weiner, 1992; North Korea
8	Head with 2+2 dorso-medial chaetae, mandible with 5–6 teeth, vestigial furca with 1+1 chaetae	9
–	Head with 5+5 dorso-medial chaetae, mandible with 4 teeth, tibiotarsi III with 17 chaetae, vestigial furca with 2+2 chaetae	*Caputanurina serrata* Lee, 1983; South Korea
9	Tibiotarsi I–III with 18, 18, 17 chaetae, mandible with 5 teeth, ant. III with ventral guard sensillum inserted on an integument swelling, maxillary external lamella long with bent apex	*Caputanurina major* Najt & Weiner, 1992; North Korea
–	Tibiotarsi I–III with 19, 19, 18 chaetae, mandible with 6 teeth, ant. III with ventral guard sensillum not inserted on an integument swelling, maxillary external lamella long with 7–10 teeth	*Caputanurina sexdentata* Najt & Weiner, 1992; North Korea

## Supplementary Material

XML Treatment for Leenurina

XML Treatment for Leenurina khualaza

XML Treatment for Leenurina pomorskii

## References

[B1] Cassagnau P (1974) Chétotaxie et phylogénie chez les Collemboles Poduromorphes. Pedobiologia 14:300-312

[B2] Deharveng L (1983) Morphologie évolutive des Collemboles Neanurinae, en particulier de la lignée néanurienne. Travaux du Laboratoire d’Ecobiologie des Arthropodes édaphiques de Toulouse 4 (2):1-63

[B3] Kuznetsova N (1988) Family Neanuridae. In: Chernova NM, Striganova BR (Eds) Collembolan fauna of USSR. Nauka, Moscow, 101–132.

[B4] Lee B-H (1983) A new subfamily Caputanurinae with two species of neanurid Collembola from Korea and the evolutionary consideration. The Korean Journal of Entomology 13 (1):27-36

[B5] Najt J Weiner WM (1992) *Koreanurina* new genus, *Leenurina* new genus, and *Caputanurina* Lee, 1983 (Collembola: Neanuridae) from North Korea. The Pan-Pacific Entomologist 68 (3):200-215

[B6] Wu DH Yin WY (2007) New record of the genus *Caputanurina* Lee, 1983 (Collembola: Neanuridae) from China, with description of a new species. Zootaxa 1411:43-46

[B7] Yosii R (1960) Studies on the Collembolan Genus *Hypogastrura*. The American Midland Naturalist 64 (2):257-281 doi: 10.2307/2422661

